# Electrical brain stimulation induces dendritic stripping but improves survival of silent neurons after optic nerve damage

**DOI:** 10.1038/s41598-017-00487-z

**Published:** 2017-04-04

**Authors:** Petra Henrich-Noack, Elena G. Sergeeva, Torben Eber, Qing You, Nadine Voigt, Jürgen Köhler, Sebastian Wagner, Stefanie Lazik, Christian Mawrin, Guihua Xu, Sayantan Biswas, Bernhard A. Sabel, Christopher Kai-Shun Leung

**Affiliations:** 1 Institute of Medical Psychology, Center for Behavioral Brain Sciences, Otto-von-Guericke University, Magdeburg, Germany; 2 Institute of Statistics, Magdeburg-Stendal University of Applied Sciences, Magdeburg, Germany; 3 Institute of Neuropathology, Otto-von-Guericke University, Magdeburg, Germany; 4 Department of Ophthalmology and Visual Sciences, The Chinese University of Hong Kong, Hong Kong, P.R. China; 5 Department of Emergency Medicine, Emory University, Atlanta, USA

## Abstract

Repetitive transorbital alternating current stimulation (rtACS) improves vision in patients with chronic visual impairments and an acute treatment increased survival of retinal neurons after optic nerve crush (ONC) in rodent models of visual system injury. However, despite this protection no functional recovery could be detected in rats, which was interpreted as evidence of “silent survivor” cells. We now analysed the mechanisms underlying this “silent survival” effect. Using *in vivo* microscopy of the retina we investigated the survival and morphology of fluorescent neurons before and after ONC in animals receiving rtACS or sham treatment. One week after the crush, more neurons survived in the rtACS-treated group compared to sham-treated controls. *In vivo* imaging further revealed that in the initial post-ONC period, rtACS induced dendritic pruning in surviving neurons. In contrast, dendrites in untreated retinae degenerated slowly after the axonal trauma and neurons died. The complete loss of visual evoked potentials supports the hypothesis that cell signalling is abolished in the surviving neurons. Despite this evidence of “silencing”, intracellular free calcium imaging showed that the cells were still viable. We propose that early after trauma, complete dendritic stripping following rtACS protects neurons from excitotoxic cell death by silencing them.

## Introduction

Non-invasive electrical brain stimulation is a new therapeutic option for the treatment of central nervous system disorders. It does not have the risks associated with surgery and has been shown to improve neurological and ophthalmological functions in normal subjects and in patients with different CNS disorders^1–10^. Specifically, repetitive transorbital alternating current stimulation (rtACS) improves visual performance after optic nerve damage as demonstrated in several clinical trials, and it has significant effects in experimental animal studies^11–16^. For example, when rodents were treated with rtACS immediately after an optic nerve crush (ONC), significantly more retinal ganglion cells (RGCs) survived than in sham treated controls^17^. In addition, the usual early post-ONC swelling and late, pre-death shrinkage of RGCs were reduced by rtACS treatment^18^. However, in contrast to the successful restoration of function in chronic disease state, electrical stimulation applied immediately post-ONC did not induce functional improvement, as evaluated by behavioural brightness discrimination test and visual evoked potentials (VEPs) in rats^12, 17^.

But such functional impairments may not necessarily be caused simply by cell death but also by functional “silencing” of cells which otherwise survive, a phenomenon which can be recognized in the work of several groups^19, 20^. Such silent survivor neurons fall into one of two categories depending on their distance to the lesion: (i) remote cells which are not directly damaged but their activity is inhibited and (ii) cells which are structurally damaged but still manage to survive. In previous studies we and others have shown that the first type (i) “remote” neurons, are silenced by inhibition and they can be re-activated by electrical stimulation or by reducing the activity of inhibiting structures^14, 21, 22^. In the experiments we now performed the neurons (RGCs) also survived but directly suffered damage by traumatic axon injury (ONC). This is compatible with the second type (ii) of survivor cells. However, in contrast to the effect of brain stimulation on inhibited, silent neurons, the impact of electrical stimulation on structurally damaged, silent neurons is largely unexplored. Therefore the aim of the present study was to elucidate the mechanisms whereby the RGCs may survive despite impaired function (“silent survivors”) and to determine the influence of rtACS treatment on the RGCs survival and functioning.

To this end we quantified neuronal survival following ONC in rodents, monitored the size of RGCs *in vivo* and analysed the level of neuronal activity using a fluorescent marker of intracellular free calcium^23^. In addition, we assessed *in vivo* dendritic arborisation of RGCs expressing a fluorescent protein in the retinae of Thy1-YFP mice^24^. Dendritic regression is often seen as a sign of neurodegeneration or ageing^25–28^. One might predict therefore that rtACS-induced neuroprotection might be associated with a preserved dendritic tree morphology post ONC. However, loss of dendrites and re-growth are also present when neuronal plasticity unfolds^28–31^ and therefore may not necessarily be a manifestation of cell death but rather one of reorganization and cell survival. As we now report, rtACS leads to massive pruning of the dendritic branches in RGCs after optic nerve crush, but this enables RGCs survival via the mechanism of functional silencing.

## Results

To reveal the effects of rtACS, rodents were subjected to different treatment schedules: ONC/SHAM animals were lesioned and not stimulated, in the ONC/rtACS groups animals received transorbital electrical stimulation after ONC, SHAM/SHAM animals received neither lesion nor stimulation and SHAM/rtACS were without crush but stimulated with rtACS.

### Traumatic Damage and Effects of rtACS-induced Protection

#### Optic nerve damage

**Figure 1:**
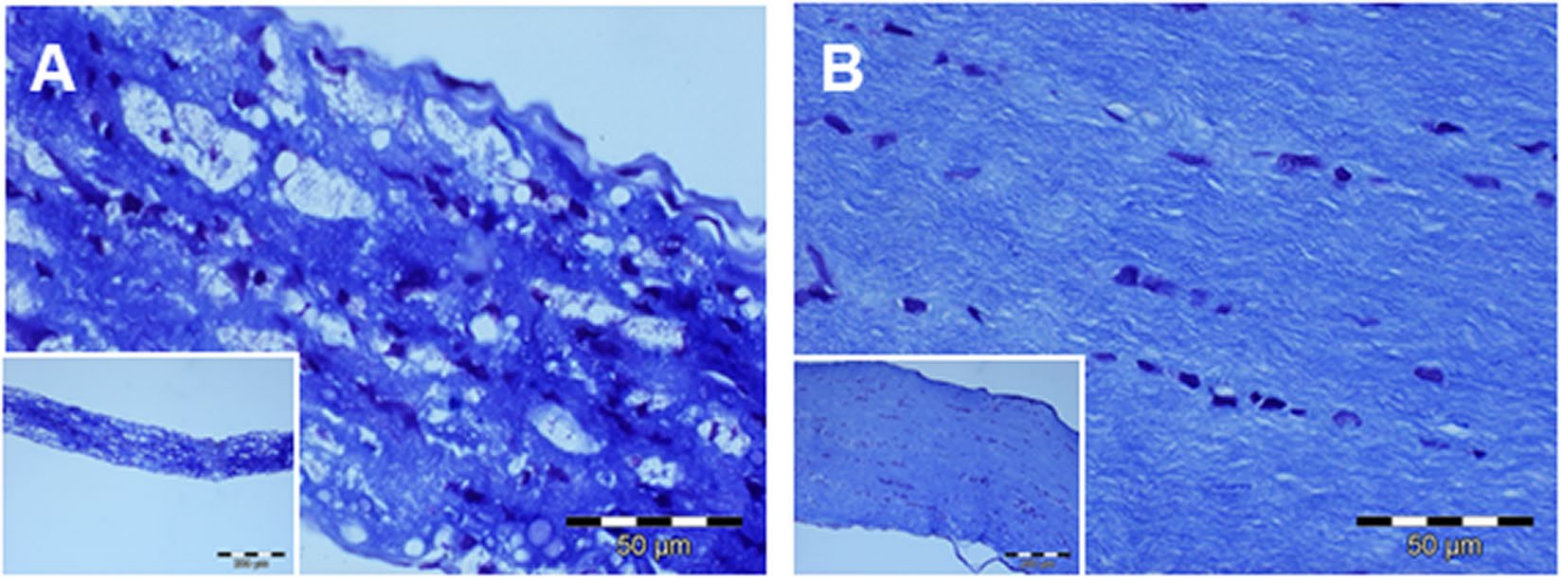
Histology of the intact and damaged optic nerve. In the photomicrographs provided in Fig. 1 illustrate the impact of severe ONC on the rat’s optic nerve: in contrast to a control nerve from a non-lesioned rat as shown in (**B**), a massively reduced diameter of the lesioned nerve can be recognized in (**A**) and a clearly damaged structure.

The impact of the severe crush on the rats’ optic nerves was revealed by histological staining with Luxol-fast-blue: the slice of nerve tissue from a sham-operated rat (no crush; Fig. 1B) looks well-structured and with normal width whereas the nerve from a rat post ONC (Fig. 1A) is clearly thinned, with vacuoles and no properly aligned morphological structures. This is in line with results from former experiments which demonstrated a significant thinning of the nerve after ONC but no difference between ONC/SHAM and ONC/rtACS^12^.

### ONC leads to delayed neuronal death and rtACS improves survival

We performed retinal imaging with *in vivo* confocal neuroimaging (ICON) technique using a confocal laser scanning microscope. The *in vivo* imaging enabled us to examine the retina in living animals repeatedly and with cellular resolution. The RGCs had been labelled via retrograde axonal transport after intracerebral injection of a fluorescent dye into the superior colliculi (where the majority of optic nerve axons project) 11 days before the imaging experiments started. The ICON technique allows following individual neurons and quantifying the number, size and fluorescence intensity of RGCs^32–35^. However, ICON is limited to the characterisation of the cell soma, as the dendrites are not labelled by this procedure.

In line with literature^36^ and our earlier studies^17, 18, 33, 35^, optic nerve damage induced dying-back pathology resulting in a delayed neuronal death. Therefore almost no loss of neurons was detected in the ONC/SHAM group and in the ONC/rtACS group on first data acquisition point, i.e. day 4 after the crush (average percentage of surviving neurons (=survival): ONC/SHAM 98.0 ± 2% from baseline versus ONC/rtACS 98.8 ± 0.8%; mean ± SEM; p = 0.9; two-tailed Mann-Whitney U-test).

**Figure 2:**
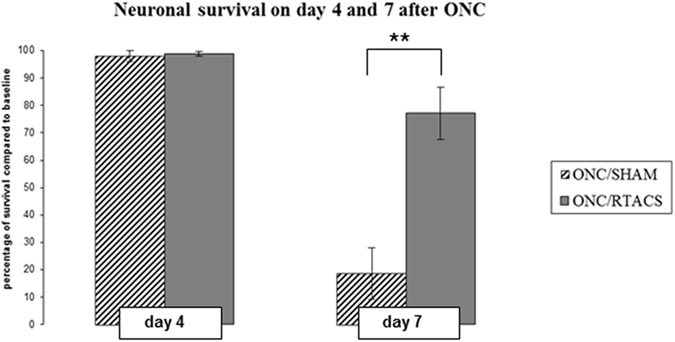
Neuronal survival on day 4 and 7 post ONC with and without stimulation. This bar graph demonstrates the significant effect of rtACS on neuronal survival on day 7 post ONC. Whereas on day 4 post ONC almost no reduction in cell number was detected in both groups (ONC/SHAM and ONC/rtACS) and no statistical difference between the groups was detected, on day 7 post ONC a significantly higher percentage of surviving neurons was observed in the ONC/rtACS compared to the ONC/SHAM group.

**Figure 3:**
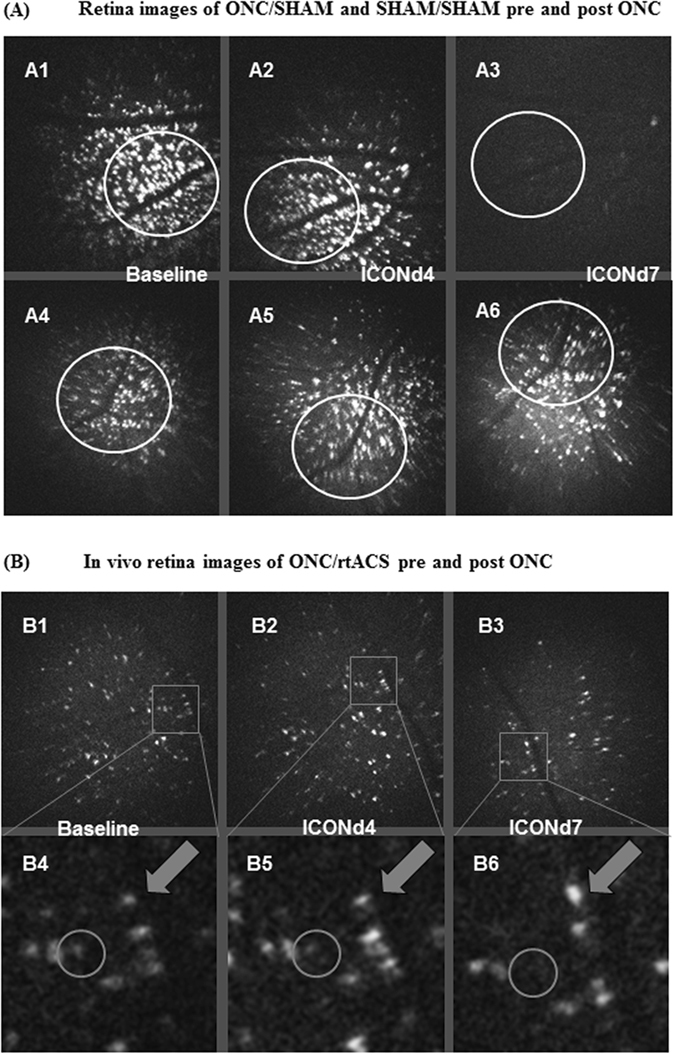
*In vivo* confocal neuroimaging (ICON) of the retina. These microphotographs demonstrate the *in vivo* morphological changes of retinal ganglion cells after ONC. In Fig. 3A1–A3 the preserved survival of RGCs on day 4 post ONC (A2) as compared to baseline (A1) is exemplified. The significant cell death in ONC/SHAM animals between day 4 and day 7 can be revealed when matching the images (A2) and (A3). The photomicrographs (A4), (A5) and (A6), showing the ICON results on baseline, day 4 post sham ONC, day 7 post sham ONC, indicate that in unlesioned rats no loss of RGCs developed within this period. The circles outline identical regions imaged at the different time points. Panel B displays the same region of the retina of an ONC/rtACS-treated animal﻿, which was imaged repeatedly at several data acquisition points, i.e. before ONC (baseline; B1, B4), on day 4 (B2, B5) and day 7 post-ONC (B3, B6). The circles in the detailed images (B4), (B5), (B6) outline a single RGC with a rather weak fluorescence intensity which disappeared on day 7 after the crush. In contrast, the arrows in (B4), (B5), (B6) point to a cell which survived and which had an increased soma size at 7 days after ONC.

However, on day 7 post ONC the degeneration of RGCs was clearly visible with survival of only 18.6 ± 9.5% in the ONC/SHAM group, whereas the ONC/rtACS group showed a significant protection of 77 ± 9.4% survival (mean ± SEM; **p = 0.01, two-tailed Mann-Whitney U-test; (see Figs 2 and 3)).

As described previously^34, 37^, SHAM/SHAM animals did not show any obvious changes in the RGC number over time (Fig. 3A).

### Visual evoked potentials were abolished in ONC/SHAM and ONC/rtACS animals

**Figure 4:**
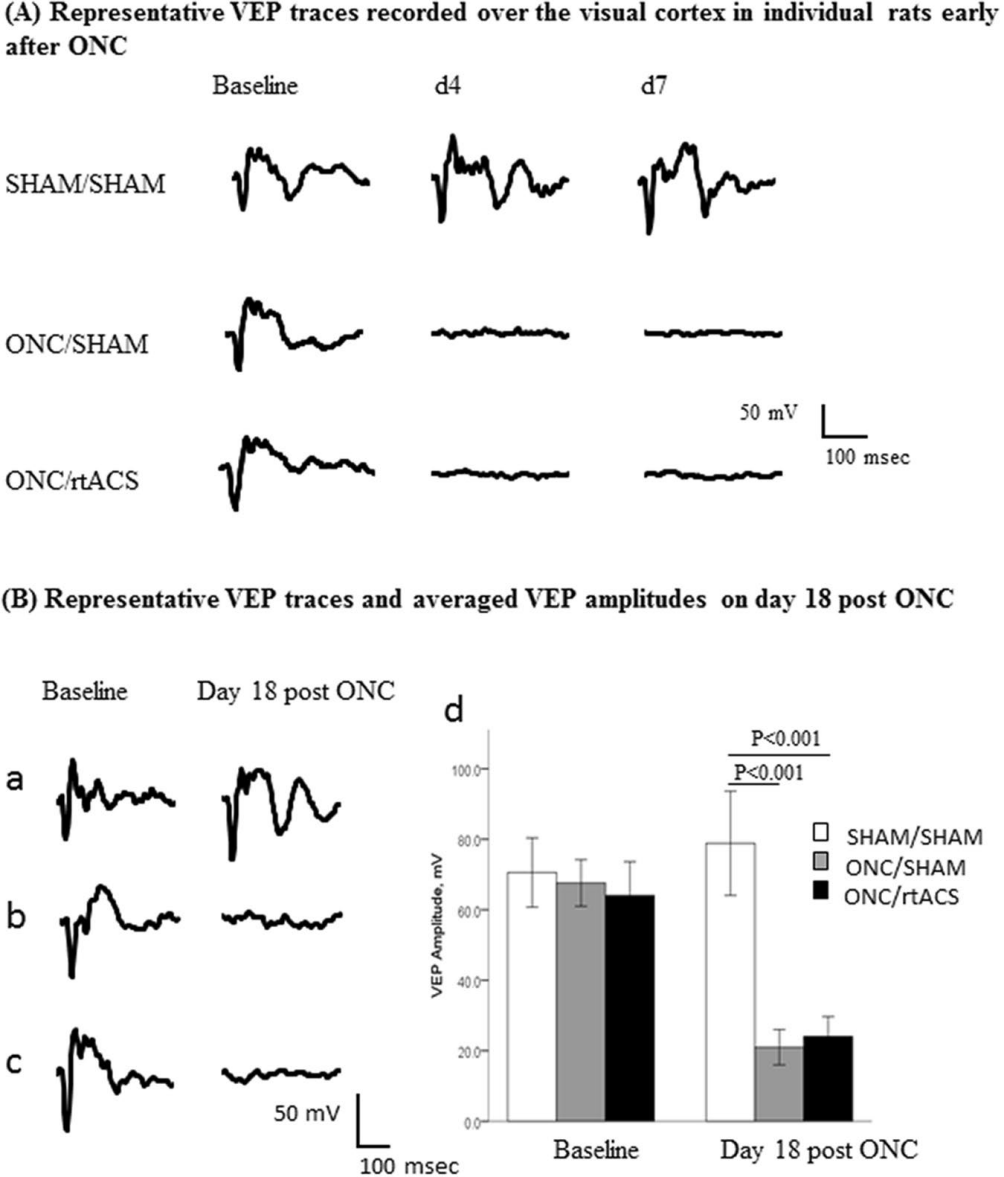
Impairment of signal transduction after ONC. In (**A**) the disappearance of the VEPs at early post-ONC time-points, i.e. day 4 and day 7 is illustrated. The examples if single traces in the left panel in (**B**) exemplify the disappearance of the VEP on day 18 post ONC. In addition, the bar graph in (**B**) represents the group values of VEP’s peak-to-peak amplitude and demonstrates the significant loss of visual function on day 18 post ONC, regardless of the rtACS treatment. The potentials from V1 contralateral to the stimulated eye are shown.

We confirmed results from our former work^12^ by demonstrating the loss of visual evoked potentials (VEPs) in animals as early as day 4 post ONC, a time-point when virtually no cell death was found. There was no functional recovery on day 7 post lesion since the VEP peaks in both groups were not detectable as on day 4 (Fig. 4A). Even 18 days after ONC the recorded signals looked like on day 4 and 7: statistical analysis revealed that VEP amplitudes were reduced significantly in both lesioned groups (SHAM/SHAM vs. ONC/rtACS (p < 0.001), SHAM/SHAM vs. ONC/SHAM (p < 0.001)) and functional recovery was observed neither in the ONC/SHAM group nor in the rtACS treated group (ONC/SHAM vs. ONC/rtACS; p = 0.592). In Fig. 4B the difference in the average peak-to peak amplitude between 3 groups is shown.

### Morphology and Mechanisms

#### rtACS prevents pre-death shrinkage and large neurons benefit most

At day 4 post ONC (ICONd4) more than 98% of neurons were alive in both groups, i.e. ONC/SHAM and ONC/rtACS. At that time-point we did not detect any significant differences in average soma size when comparing both groups as well as no significant difference within each group when comparing ICONd4 measurements to baseline values. However, when selectively measuring the shrinking neurons in each group, i.e. decreasing soma size from baseline to ICONd4 (a sign of degeneration^28^), an effect of rtACS was revealed: the shrinkage in ONC/SHAM group was significantly more pronounced than in the ONC/rtACS group (difference in soma size (pixel units) from ICONd4 to baseline in group ONC/SHAM: −285.2 ± 29.9 versus difference from ICONd4 to baseline in group ONC/rtACS: −154.2 ± 24.3; **p < 0.01; Student’s t-test).

The average soma size of the neurons measured at ICONd7 in the ONC/rtACS group was slightly (17%) but significantly larger than their baseline value (baseline ONC/rtACS: 447.2 ± 40.0 versus ICONd7 Survivor ONC/rtACS: 527.2 ± 51.7; *p = 0.03; paired t-test). The surviving neurons at ICONd7 in the ONC/SHAM group did not show any significant difference compared to baseline.

With ICON, individual neurons were followed repetitively over time, and their “fate” in reference to their baseline values was traced. This allowed categorising the baseline population of neurons into “will survive until ICONd7” and “will be dead at ICONd7”. Thus when the size of these two categories of neurons was compared in rtACS-treated animals, at baseline the later “survivors” were the significantly larger cells than the neurons which 7 days afterwards were found to be dead (baseline of survivor (ONC/rtACS): 447.2 ± 40.0 versus baseline of doomed RGCs (ONC/rtACS): 230.2 ± 29.7; ***p < 0.001; Student’s t-test; exemplified in Fig. 3B). There was no significant baseline difference between day 7 survivors and non-survivors detected within in the ONC/SHAM group. This finding suggests that rtACS treatment protected mostly larger RGCs.

#### Dendritic Stripping after rtACS treatment in mice following ONC

To evaluate the effect of rtACS on sub-cellular morphology of the dendritic tree after ONC we performed *in vivo* ophthalmologic imaging utilizing Thy-1-YFP16Jrs mice where the expression of YFP corresponds well to the dendritic structure even when the dendritic tree is shrinking, which was confirmed in our previous publications^38, 39^. However, the fluorescent protein is expressed only in less than 1% of RGCs. Because of such a restricted labelling, the model is not well suited for the assessment of cell numbers. Nevertheless, even taking into account this limitation, we could demonstrate that our crush method led to the typical ONC-induced delayed cell death in the Thy-1-YFP16Jrs mice. In this experiment a rather mild lesion was induced and, as expected, on day 3 post ONC the imaging did not reveal any neuronal decline. On day 7 post ONC amount of RGCs was slightly decreased (percentage of survival ONC/SHAM: 77.1 ± 7.1; ONC/rtACS: 88.0 ± 17.1), yet unlike in the rat study the difference between groups was not significant. However, 14 d after the crush significantly fewer neurons were found in both the ONC/SHAM and the ONC/rtACS group as compared to SHAM/rtACS (surviving neurons as percent of baseline): ONC/SHAM: 53.6 ± 4.5% vs ONC/rtACS: 55.3 ± 3.7 vs SHAM/rtACS 96.7 ± 8.9; ***p < 0.001; ANOVA).

**Figure 5:**
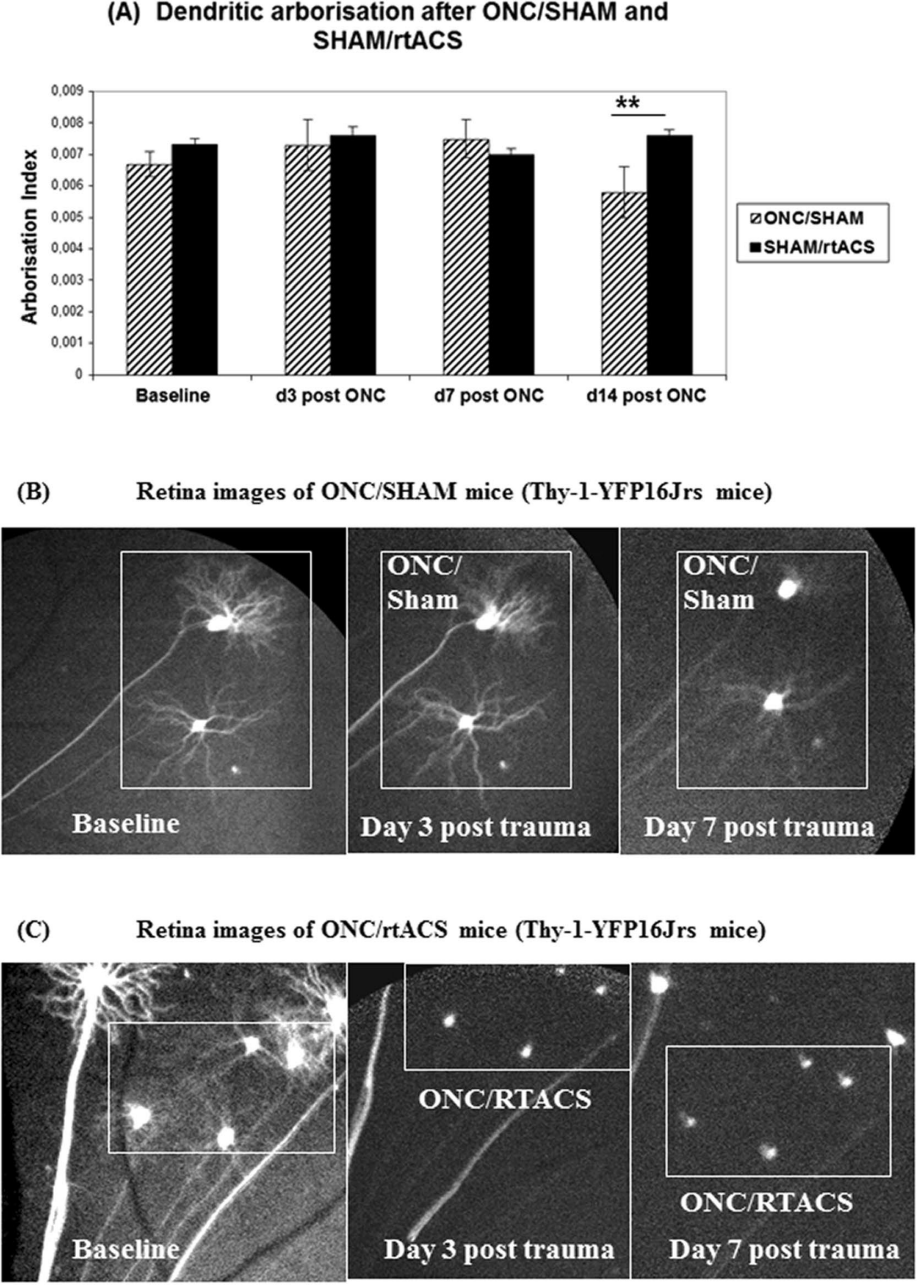
Influence of axonal trauma and rtACS on dendritic morphology. In (**A**) the bar graphs representing the dendritic tree arborisation index demonstrate the dendritic regression in the ONC/SHAM group at 2 weeks after ONC as compared to non-lesioned controls (SHAM/rtACS). Also of note is that no data could be collected from the lesioned, rtACS stimulated animals as no dendrites were visible after ONC in the ONC/rtACS group. (**B**) and (**C**) illustrate the differences in dendritic arborisation after ONC depending on the treatment schedule. Whereas RGCs with SHAM stimulation exhibit a clearly visible dendritic tree early after ONC (Fig. 5B), in the ONC/rtACS group only the soma without any dendrites was detectable even at the earliest time point of imaging (3 post ONC; (**C**)).

Regarding the dendritic tree morphology Sholl analysis revealed no significant differences in the arborisation index at the early time points (day 3 and 7 post ONC) between the ONC/SHAM and the SHAM/rtACS group. But by the time cell survival declined, i.e. on day 14 post ONC, a significant reduction in the arborisation index became apparent (ONC/SHAM: 0.0058 ± 0.0008 vs SHAM/rtACS 0.0077 ± 0.0002; **p < 0.01: Student’s t-test; Fig. 5A,B). Interestingly, however, in the ONC/rtACS group, no neurons with visible and measurable dendritic trees were detected on day 3, day 7, and day 14, although soma were still present (Fig. 5C).

### Moderate increase in fluorescence intensity indicates viability of rtACS-treated surviving neurons

Quantification of fluorescence intensity of the neurons labelled with Oregon Green BAPTA (an indicator of intracellular free calcium) revealed no significant difference between the groups at any post ONC data acquisition time point. However, there was a slight, but significant increase in fluorescence intensity in the ONC/rtACS group when comparing the surviving neurons at ICONd7 with their baseline values (grey values ONC/rtACS baseline: 106.8 ± 9.8 versus ONC/rtACS ICON7d: 128.0 ± 12.9; **p = 0.016; two-tailed Student’s t-test). Interestingly, in this treated group we also observed a difference in the baseline fluorescence values between neurons which survived and those which were dead by day 7 (ONC/rtACS group grey values baseline of survivors: 106.8 ± 9.8 versus baseline of doomed 70.3 ± 11.7; **p = 0.018; two-tailed Student’s t-test).

## Discussion

Standard analysis of brain damage and protection usually defines tissues either as healthy or damaged and neurons as either alive or dead. However, our research and that of others laboratories propose the need for a more refined concept of cell death and survival because a treatment can *increase* cell death yet *improve* brain function^40^, post-lesional re-structured networks of surviving neurons can lead to dysfunction^41–43^, a very defined, local lesion can lead to very complex functional deficits^44^ and, as shown in our studies, surviving neurons may become “silent”^17, 28^.

Here we demonstrate that this “silent” state of injured neurons is not necessarily an irrevocable predictor of cell death because rtACS rescued silent RGCs post lesion. This improved survival is in line with our prior results and compatible with the findings reported by others^17, 18, 45–50^.

The analysis of dendritic tree morphology, though, revealed an unexpected mode of rtACS action: in non-treated lesioned mice (ONC/SHAM group) the RGCs’ dendritic tree was slowly disintegrating in a delayed fashion and was paralleled with increased cell death. Similar decline of dendritic tree after ONC has been shown also by other laboratories^51^. However, in the ONC/rtACS group the dendritic tree was completely abolished already at the first imaging time point, on day 3 post ONC. We hypothesise that via this dendritic pruning rtACS can induce an “input-silencing”: in addition to the interrupted axonal signal transmission, excitation of the neurons was prevented by fast and complete pruning of their dendritic trees. At first sight it seems counterintuitive that this additional “destruction” of dendritic morphology may be associated with neuroprotection, as demonstrated by increased neuronal survival in the ONC/rtACS group compared to ONC/SHAM. However, we suggest that the dendritic stripping might, in fact, isolate RGCs from incoming signals, and thus protect them from excitotoxic cell death^52–54^. Importantly, these stimulation effects of rtACS seem to exclusively affect neurons after damage as we did not see any decline of the dendritic tree in the unlesioned control mice treated with rtACS. It can therefore not be interpreted as a “toxicity” effect of stimulation.

As a consequence of this protective elimination of dendrites the significant post-lesion shrinkage of the neurons’ soma may be prevented. Such reduction in size happened in the ONC/SHAM group and is a hallmark of RGC degeneration^18^. In contrast, with our repeated *in vivo* imaging we were able to demonstrate that the surviving neurons were slightly but significantly increased in size (17%) after ONC/rtACS compared to baseline, but this moderate swelling was clearly smaller than the characteristic swelling of an acute toxic oedema^55, 56^. Therefore we suggest that this moderate swelling is a sign of long-term survival or neuronal plasticity^51, 57^ and not an indicator of future cell death.

Repeated ICON measurements in each animal before and after ONC also allowed us to determine the future fate of neurons based on their pre-ONC morphological characteristics. The comparison of RGCs’ size of survivors/non-survivors in the ONC/rtACS group at baseline revealed that rtACS seems to be especially beneficial for the larger size neurons: they survived significantly better than small-sized neurons which were rather doomed to die. One explanation of this finding may be that the parasol subtype of RGCs cells – which is relatively large as compared to other subtypes - survived better than the smaller midget cells. However, the number of parasol cells in proportion to the total number of neurons is actually too low to explain the difference in the overall cell survival^58, 59^. In fact, a possible size-dependent vulnerability of RGCs is discussed quite controversially^60^. The *in vivo* imaging method (ICON) does not allow distinguishing between the RGCs subtypes; hence morphological classifications can currently not be determined. However, our results of a slightly but significantly elevated free intracellular calcium level at baseline in the survivor neurons as compared to non-survivors, is in line with the hypothesis that rather the functional condition and not the morphological classification is decisive for survival. In addition, the finding that fluorescent calcium signal was slightly but significantly greater on post ONC day 7 than at baseline in the rtACS group indicates that the stripped cells are still viable and that they are undergoing active processes of cellular post-lesional plasticity^23^.

However, our study addressed only a rather short post-lesion period to detect early rtACS-induced mechanisms of silencing. Therefore we cannot exclude the possibility that the protective “structural anti-excitotoxic” effect of rtACS during the early stage following traumatic pathology may later expose the neurons to a delayed cell death. Our earlier studies suggest that though cell death is continuing after day 7 post ONC^18^ even in rtACS-treated animals, there still may be beneficial long-term effects of stimulation on survival^17^. In addition, rare cases of long-term survival of retinal ganglion cells with impaired dendritic trees after ONC without stimulation treatment have been demonstrated. These cells were also stripped off dendrites and had damaged axons but the soma was still detectable up to 133 days post ONC^39^. Further studies should focus on the “midterm” (sub-acute) and long-term post-traumatic periods (2–4 weeks after ONC and longer) and investigate whether repeated rtACS can sustain neuronal survival and whether regrowth of the dendritic processes and regeneration of axons – and as a consequence restoration of function - is possible for silent survivors. For example, pharmacological promotion of dendritic branching and functional recovery has been demonstrated^61^.

Taken together, our results suggest that excitotoxic cell death may thus be interrupted by rtACS-induced dendritic pruning.

However, the question remains how electrical stimulation activates this intrinsic “functional defence” mechanism against cell death. Dendritic pruning is widely investigated in the area of developmental biology. Here inhibition and lack of activation leads to retraction of unused branches, which is the basis for neuronal system refinement^62, 63^. However, dendritic pruning has also been described as a consequence of brain lesions^64^, though it seems that the mechanisms are quite different in this case and more similar to effects of inflammation and glutamate-mediated excitation^65, 66^. Experimental studies in adult animals demonstrated that the development of dendritic pruning after brain lesions is delayed^67^, which is in accordance to what we detected in the control animals (ONC/SHAM group). This not-treated/not-influenced, late dendritic pruning may be associated with apoptotic pathways^68^.

Strong electrical stimulation can per se also lead to dendrite damage. In the early studies of long-term potentiation, the protocols with high electrical currents led to dendritic damage^69^. Interestingly, this damage appeared rather fast, as we also see in our experiments under rtACS conditions, and it seemed to be mediated by excitotoxicity. In the present study, however, we used rather low current amplitudes and no tetanic stimulation. As expected, these conditions did not induce any damage in intact animals (SHAM/rtACS group). However, our former work demonstrated that dying RGCs increase intracellular free calcium following ONC, i.e. suffer excitotoxicity^23^. On the other hand, a low frequency electrical stimulation was shown to increase intracellular calcium levels^70^. We therefore propose that the dendritic pruning which we observed in stimulated animals with ONC, might be mediated by toxic levels of calcium due to an additive effect of lesion-induced and stimulation-induced calcium rise. Interestingly, a recently published study reported that dendritic degeneration occurred within 6 hours in a retinal explant model, whereas cell death was not observed until day 14^71^. This finding is in line with our results and the hypothesis that in general additive challenges - like axotomy and artificial *in vitro* conditions in the retinal explant model - may lead to fast dendritic pruning and by this mechanism assure survival of neurons. The current finding is also in agreement with our previous study demonstrating that the NMDA-antagonist MK801 leads to increased cell death after ONC^40^. As we hypothesize that a fast post-lesional dendritic pruning protects cells from further excitotoxic threat, acute MK801 application may prevent lesion-induced pruning but leave the neurons vulnerable to delayed death due to excitotoxicity.

However, in order to propose an additive effect of excitotoxicity and electrical stimulation it is important to know whether the electrical current applied actually reaches the RGCs. Modelling current flow for rtACS experiments in humans (stimulating electrode on the forehead above the eye brow) suggests that most of the current enters the brain via the eye because the high percentage of fluid in this structure is the most conductive medium^72^. As in our animal experiments the gold ring electrode was placed directly onto the cornea, we assume that virtually all the current is injected via the eye to the retina/optic nerve and preliminary data using a phantom model (supplementary data) suggest that current distribution/intensity in a homogenous gel (similar like in the eye) follows the basic, resistance-length-dependent law. These modelling data and evidences from Foik *et al*. (2015) confirm that rtACS stimulates retinal neurons^73^.

The considerations based on the electrical activation of retinal neurons still raise a question whether photic stimulation yields comparable results. To the best of our knowledge, there are no studies published describing the influence of rhythmic photic stimulation on the survival of retinal ganglion cells after an injury. However, there are few papers demonstrating neuroprotective effects of transcorneal electrical stimulation after optic nerve transection^46–49^ or optic nerve crush, including our works^17, 74^. Interestingly, electrical brain stimulation and photic stimulation have been compared in terms of their effects on brain activity. While both alternating current stimulation and rhythmic visual stimulation can synchronize ongoing brain oscillations^75, 76^, electrical stimulation yields aftereffects outlasting the period of stimulation compared with photic stimulation^15, 77^. Although the modifications of brain oscillations might be hardly extrapolated to the retina, one can suggest that similar to the persisting synchronization in the brain networks, electrical stimulation can induce prolonged aftereffects in the retinal networks. Moreover, the retina would likely need to be functional - which is not the case following retrograde degeneration after the optic nerve crush - in order for photic stimulation to take the effect. In contrast, electrical pulses to the eye can directly stimulate not only photosensensitive cells, i.e. photoreceptors, but all the retinal layers, including retinal ganglion cells, as well as the optic nerve fibers^72, 73^. Therefore it is plausible that electrical stimulation may outperform photic stimulation in the injured retina/optic nerve. Additional studies would be needed to address this important question.

Taken together, our study shows that understanding the post-lesion neuronal state requires concepts beyond the categories “life or death” of neurons and that rtACS can promote survival of neurons yet functionally silence them. “Silent survivor cells” and dendritic stripping are the novel factors to consider for understanding the full spectrum of post-traumatic plasticity and may help uncover the mechanisms of vision restoration and recovery.

## Material and Methods

**Figure 6:**
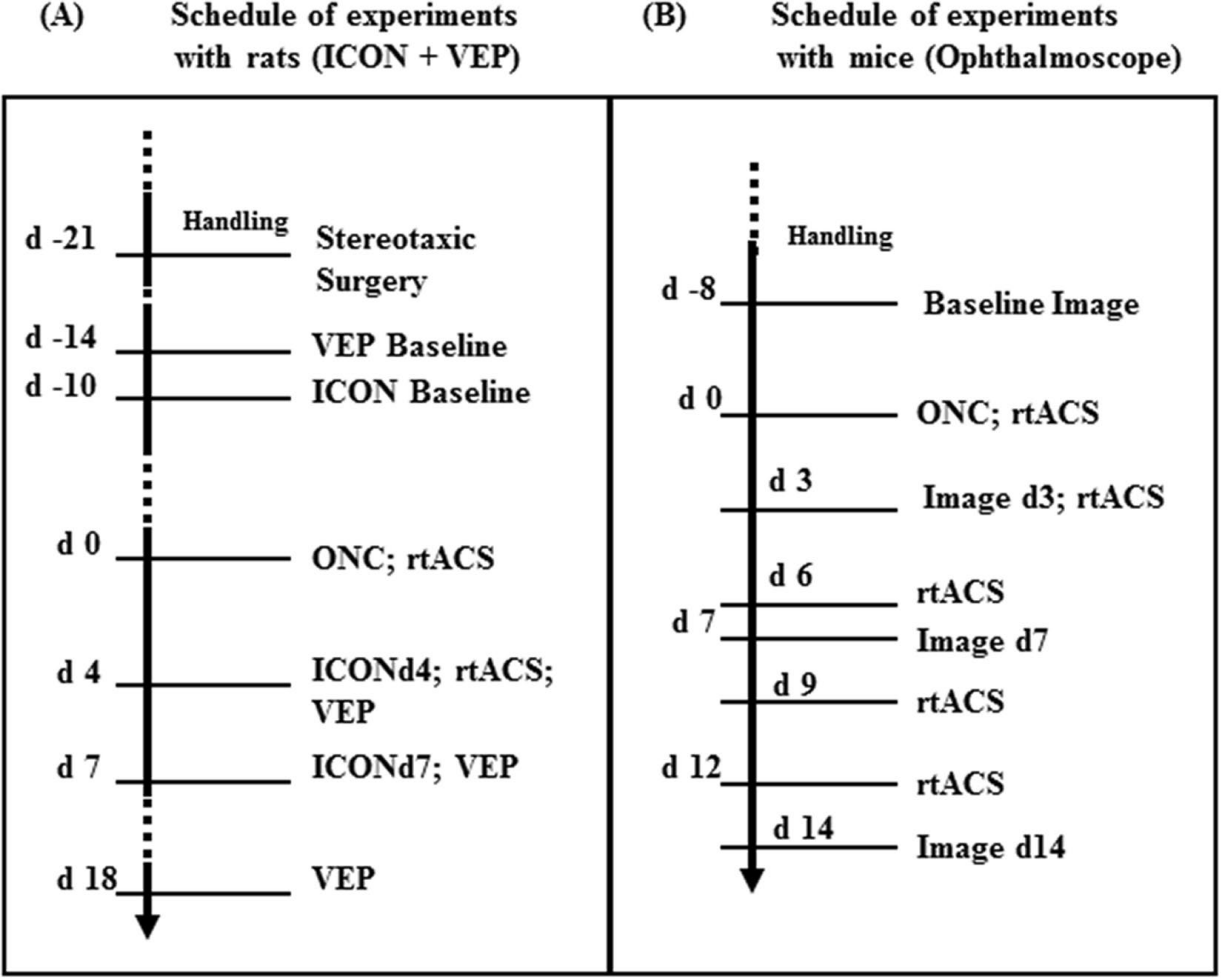
Schedule of experiments. This scheme provides an overview of the two experimental schedules utilizing (i) rats for ICON and VEP experiments (left scheme **A**) and utilizing (ii) transgenic Thy1-YFP-mice for retina imaging with ophthalmoscopy (right scheme **B**).

For an overview of experimental schedules see Fig. 6.

### Experiments and methods utilized in rats

#### Experimental schedule for In vivo Confocal Neuroimaging (ICON), Optic Nerve Crush (ONC) and Visual Evoked Potentials (VEPs)

Male hooded rats CrL: LIS obtained from Charles River, 6-7 weeks of age were kept at 12-h light/dark cycle, ambient temperature 24–26 °C and humidity of 65%. Prior to the experiment the rats were adapted to the new environment and handled for 3–5 days. Food and water were available ad libitum.

For retrograde labelling of RGCs^35^, animals’ superior colliculi were injected stereotaxically with fluorescent marker Oregon Green BAPTA 21 days before ONC. In addition, electrodes to record visual evoked potentials (VEPs) were implanted into visual cortices. After recovering from surgery and 10 days before crush a baseline ICON^32^ was taken. The animals were then randomly assigned to one of the three groups: (i) ONC^78, 79^ with subsequent rtACS (ONC/rtACS, n = 11)^12, 15^, (ii) ONC with sham stimulation (no current flow through electrode; ONC/SHAM, n = 8) and (iii) transient exposure of the optic nerve but no crush with sham rtACS (SHAM/SHAM, n = 9). The ONC and the first rtACS stimulation were done on day zero (experimental schedule, see Fig. 6A). ICON was performed on day 4 (ICONd4) whereafter the animals received rtACS or sham stimulation. On day 7 post ONC another ICON was performed (ICONd7). In the SHAM/SHAM group we performed ICON analysis in two animals to confirm the previously established stability of labelling^2, 17, 18, 23^.

The early loss of visual function, as shown already by Sergeeva *et al*.^12^, was corroborated by measuring VEPs in single animals at the days of ICON (d4 and d7). As a follow-up, VEPs were recorded in all three groups at day 18 post ONC. The experimental schedule is illustrated in Fig. 6.

Narcosis for intracerebral injection, ICON and VEPs was induced with ketamine (75 mg/kg, i.p.) and xylazine (10 mg/kg, i.p.).

For all procedures ethical approval was obtained according to the requirements of the German National Act on the use of experimental animals (approved by Landesverwaltungsamt Sachsen-Anhalt; AZ 42502-2-942 UniMD) which is in accordance with the Directive 2010/63/EU).

#### ICON experiments

Retrograde labelling of RGCs was induced by injecting 2 µl Oregon Green BAPTA (10% in phosphate buffered saline with 0.1% DMSO) into both superior colliculi. To this end, bore holes were drilled into the skull and intracerebral injections were placed with a Hamilton syringe at the following coordinates: AP: −6.9 mm, ML: 1.2 mm. The dye solution was injected at depth DV: 4.0, 3.5, 3.0 and 2.5 mm with 0.5 µl at each position.

The ICON experiments were based on previous works^23, 32, 80^ but adapted to fulfil the current experimental requirements. For ICON the rats were anaesthetized and their eyes treated with Neosynephrine-POS 2.5% (Ursapharm) to dilate the pupils. The animals were positioned on a purpose-built stage under the objective of an upright confocal laser scanning microscope (LSM 5 Pascal, Zeiss GmbH; 5x magnification) with the eye positioned underneath the objective lens. A −80 dioptre plano-concave lens (KPC-013; Newport GmbH) was placed onto the cornea (protected with Vidisic optical gel; M. Pharma) to image the retina. Three to five images were taken per ICON session covering peripheral and central parts of the retina. We used a scanning time of 24.5 s and an average of 16 scanning acquisitions. The procedure was carried out in both eyes at baseline and the eye with the best labelling was chosen for data acquisition at different time points.

The images were taken from areas containing a sufficient number of cells which could be relocated at later time points using retinal blood vessels as reference landmarks. The labeled neurons quantified at baseline, i.e. before ONC, were followed at the post-ONC data collection points and their number, size and fluorescence intensities were quantified. All evaluators were blind to the experimental conditions underlying the data sets.

The cell soma diameters were measured with the AxioVisionRel. 4.7 software (Zeiss, Jena) at different angles in each cell and averaged automatically for each cell size calculation of the soma area and expressed in pixel units (1 pixel unit corresponds to 2 µm^2^). As a consequence of the retrograde axonal dye transport only RGCs were labeled as confirmed by their morphological appearance, i.e. round, circumscribable structures clearly distinct from background. To evaluate fluorescence intensity the grey values of individual cells were recorded at different time points and compared. To quantify post-lesion survival the number of neurons at baseline before the lesions was defined as 100%. The percentage of survival was determined for each animal and averaged in each group to obtain a mean percentage of survival according to previous protocols^23, 33, 34, 80^.

#### Optic nerve crush (ONC)

To induce a traumatic lesion of the optic nerve in anaesthetised rats an incision of the conjunctiva lateral to the cornea was followed by a lateral canthotomy made from the orbita. This allowed the retractor bulbi muscles to be separated and the optic nerve to be exposed by blunt dissection. To crush the nerve a calibrated self-closing forceps (Martin Instruments, Tuttlingen) were used for 30 seconds at a distance of 2 mm from the eyeball, leaving both retinal blood supply and dura intact. The jaws of forceps stayed 0.1 mm apart in a “rest-position” which produces a severe injury^78^. After the surgery an antibiotic eye ointment (Aureomycin) was applied. ONC was performed on both eyes.

#### Stereotaxic surgery and visual evoked potentials (VEPs)

To assess visual function VEPs in rats were recorded under ketamine/xylazine anaesthesia on day 18 post ONC using Encephalan-131-03 (Medicom MTD, Russia) as previously described^12^. Traces from single animals were recorded on day 4 and 7 post lesion as well. Briefly, two stainless-steel screws with a shaft diameter of 1.17 mm (Fine Science Tools, Heidelberg, Germany) were implanted over the primary visual cortices. The signal was acquired with a sampling rate of 256 Hz, bandwidth 0–100 Hz. One hundred consecutive LED flash stimuli were presented alternating to each eye, one per sec, and the responses were averaged separately for the ipsilateral and contralateral visual cortex. Minimum and maximum values within the time window of 0–150 ms were found for every averaged VEP trace and the peak-to-peak amplitude was calculated.

To test a possible influence of different reference electrode montages (as used in the rat and mouse studies) on the stimulus artefact after rtACS, custom-made stainless steel teflon-coated recording electrodes (75 micrometers uncoated and 140 coated diameter; SS-3T/HH; Science products GmbH) were stereotaxically implanted into the primary visual cortex (AP: −7 mm; ML: 3 mm lateral; depth: 1 mm) of test rats after piercing the dura through a drill-hole in the skull and connected with pin-sockets. In addition, a screw was fixed in the occipital bone region, to be used as another reference electrode. The set-up was fixed with dental cement. Alternating current stimulation was performed as described in the next chapter via a goldring electrode with the following parameters for the test pulses: 200 µA, 40 ms, 6 Hz.

After the test animals had recovered from surgery for at least one week, rtACS was delivered after placing the reference electrode either on the tail, contralateral ear side or using the occipital screw as reference electrode. Stimulation pulses were recorded with TMS standard 8 channels porti 7 system with a sampling frequency of 2048 Hz. Data analysis revealed a significant group difference in the measured potential [µV] between the 3 groups: occip. ref: 8,973 ± 700; contralat. ref: 10,503 ± 682; tail ref: 9,995 ± 683; ANOVA; p = 0.005). Post hoc analysis revealed that signals from the occipital reference electrode were significantly different from the other two montages, but no difference between the side- and the tail placement (p = 0.22). These data indicate that different reference electrode placements on the rodent’s body (as used in our rtACS experiments) have no major influence on the transcorneal stimulation.

#### Repetitive transcorneal alternating current stimulation (rtACS)

Non-invasive electrical stimulation^12, 15^ was applied in rats immediately post ONC and on day 4 (see Fig. 6A). The stimuli consisted of biphasic square-wave pulses (pulse duration: 10 ms/phase, intensity: 200 µA) with varying frequencies similar to what has been used in patient studies and according to our former animal experiments in which we used frequencies similar to the rat’s EEG frequency dominating in a late stage of ketamine/xylazine narcosis, i.e. 5 Hz ± 3 Hz. On each day of treatment, rtACS was applied as two blocks with 120 s break between them. One block consisted of 35 cycles of bursts (15 pulses per burst, 420 pulses per cycle) of each frequency delivered alternating into both eyes in the following order: 2-3-4-5-6-7-8-8-7-6-5-4-3-2 Hz with 1 s break between bursts and 10 s break between cycles. The stimulation session lasted about 23 min.

rtACS was always applied in about 40 min after induction of ketamine/xylazine anaesthesia when preceding procedures like ONC, VEPs or ICON were completed. For the stimulation the rats received in addition local anaesthesia with Proparacain-POS 0.5%. A 3 mm diameter gold ring electrode (Roland Consult, Brandenburg) was placed on the rat´s eyes after Vidisic optical gel had been applied to the cornea to protect it from drying and to assure stable conductance between the cornea and stimulating electrode. The reference electrode was fixed on the tail. rtACS was performed with a custom-made stimulator. Sham-treated animals underwent the same procedures except that no current was applied.

#### Histology of optic nerve

At the end of the experiment the rats were deeply anaesthetized (6 ml Chloralhydrate 8%; i.p.) and transcardially perfused with 0.9% NaCl solution. The optic nerves were harvested and fixed by immersion in 4% formaldehyde solution and embedded in paraffin. Slices of 1 µm thickness were cut on a microtome and Luxol-fast-blue staining was performed. Pictures were taken with a Leica DMRXE microscope.

### Experiments and methods utilized in mice

#### In vivo imaging of dendrite morphology

The dendritic arborizations of RGCs were non-invasively imaged *in vivo* in the B6.Cg-Tg(Thy1-YFP)HJrs/J transgenic mice (The Jackson Laboratory) using a confocal scanning laser ophthalmoscope (CSLO) (HRA, Heidelberg Engineering GmbH). All mice were fed ad libitum and treated according to the use of animals in ophthalmic and vision research recommended by the Association for Research in Vision and Ophthalmology. The mice were randomly assigned to three groups: ONC/rtACS (n = 9), ONC/SHAM (n = 9), SHAM/rtACS (n = 6). The details of instrumentation and technique for *in vivo* imaging of RGCs have been described^67, 81–83^. In brief, the ophthalmoscope HRA2 with a 55 degree wide field lens was used to capture images at a rate of 16 frames/s. Image averaging (15 images) was automatically performed by the HRA2 software at the time of imaging to increase the signal-to-noise ratio. Imaging was performed by one experimenter holding the animal without systemic anaesthesia and another operating on the CSLO. Baseline images were taken 8 days before ONC. The mice were stimulated with rtACS on day 0, 3, 6, 9 and 12 post ONC and *in vivo* imaging was performed on day 3, 7 and 14.

(Experimental schedule see Fig. 6B).

#### Optic nerve crush

For ONC in mice, conjunctival peritomy was performed in the inferior limbus. The conjunctiva was gently peeled back and the optic nerve was then exposed with gentle blunt dissection of surrounding adipose tissue and blood sinuses. The optic nerve was clamped with a pair of self-closing tweezers (Dumant No. 5) for 5 seconds at 1 mm posterior to the eyeball. Topical steroid and antibiotic ointment was applied to the wound. The nerves were briefly exposed but left intact in mice subjected to sham procedure.

#### rtACS treatment

Mice were stimulated immediately after ONC surgery and on day 3, 6, 9 and 12 post ONC. Mice were anaesthetized with ketamine/xylazine and local anaesthetic Proparacain-POS 0.5% was applied to the eyes. A 2 mm diameter gold ring electrode (Roland Consult, Brandenburg) was placed on the mouse eye with Vidisic optical gel (M. Pharma) beneath for protection and better conductance. The reference electrode was fixed on the contralateral ear. Stimuli were generated by isolated pulse stimulator (A-M Systems 2100). Two 5-min sessions separated by 2-min break were applied to each eye. The session consisted of 8 consecutive 30-s trains of biphasic square-wave with pulse duration 1 ms/phase, intensity 100 µA and with frequencies 10, 12, 9, 11, 8, 10, 9, 12 Hz. Each 30-sec train was followed by 10 sec break. The stimulation session lasted about 24 min.

#### Sholl analysis

The dendritic tree was evaluated using modified Sholl analysis^38, 84^. The images from the ophthalmoscope were exported to a custom MatLab script (MATLAB R2013b, Image Processing Toolbox; The MathWorks). The grey-scale levels were transformed into black and white. The area covered by the neuron was calculated from the values of the maximum horizontal and vertical dendritic spread. The branching complexity was quantified by counting the intersection of dendrites with each of the 10 concentric circles which were drawn around the cell body with increasing radii. The sum of all intersections was set in relation to the area covered by the neuronal soma and the dendritic tree (arborisation index = sum of intersections/area [soma + dendrites]).

### Statistical analysis

Statistical analysis was performed with IBM SPSS Statistics 21 and EXCEL software. The outcome variables are presented as means and standard error of the mean. Differences in cell number evaluated by ICON were compared with two-tailed Mann-Whitney U-Test. RGC diameters (ICON) and the arborisation index (Sholl analysis) were examined with t-test for independent (group comparisons) and paired (within group trend analysis) samples. Data were tested using Shapiro-Wilk-test and QQ-Plots before further statistical analysis and no violation of normality was detected. The group differences in neuronal survival in mice and VEP amplitudes were evaluated by factorial analyses of variance (ANOVA) followed by post-hoc Bonferroni test for multiple comparisons.

A p-value < 0.05 was considered to be statistically significant.

## Electronic supplementary material

Supplementary information
