# Supplementary material for: Electrical brain stimulation induces dendritic stripping but improves survival of silent neurons after optic nerve damage
Source: Sci Rep. 2017 Apr 4;7:627. doi: 10.1038/s41598-017-00487-z (PMC5428431; doi:10.1038/s41598-017-00487-z)
Supplement: Supplementary file 1 — Supplementary information [file 41598_2017_487_MOESM1_ESM.pdf]

Supplementary data for manuscript *"Electrical brain stimulation induces dendritic stripping but improves survival of silent neurons after optic nerve damage"*
submitted to Scientific Reports

Petra Henrich-Noack*[,1,a], Elena G. Sergeeva[1,5,a], Torben Eber[1,2], Qing You[1], Nadine Voigt[1], Jürgen Köhler[2], Sebastian Wagner[1], Stefanie Lazik[1], Christian Mawrin[3], Xu Guihua[4], Sayantan Biswas[4], Bernhard A. Sabel[1], Christopher Kai-Shun Leung[4]

[1] Institute of Medical Psychology, Otto-von-Guericke University Magdeburg, Germany
[2] Institute of Statistics, Magdeburg-Stendal University of Applied Sciences, Germany
[3] Institute of Neuropathology, Otto-von-Guericke University Magdeburg, Germany
4 Department of Ophthalmology and Visual Sciences, The Chinese University of Hong Kong, Hong Kong, PRC
[5] Department of Emergency Medicine, Emory University, Atlanta, USA

[*]Corresponding author
Email: petra.henrich-noack@med.ovgu.de
Tel.: +49 391 6721806
Fax: +49 391 6721803

Current intensity mapping in a phantom model.

To address the question of current intensities in the eye after electrical stimulation we tested the electrophysical effects with a simplified phantom model, which was based on the work of Rush and Driscoll (1968).

When current is injected transorbitally via a corneal electrode, the current passes through vitrous humor gel, which occupies almost 80% of the eye and consists mainly of water with a few percent of collagen fibrils, hyaluronic acid, ascorbic acid, inorganic salts and sugar. Therefore, for our phantom model we used a simplified conducting gel, prepared of 20% gelatin in 2% propanol/water which was kept in a glass tray (size 12 x 18 cm) overnight before experiments started. Two electrodes were inserted into the conducting gel. The stimulating electrode was always in the left lower corner (1 cm away from boundaries) and currents were measured placing the second electrode at different distances from the stimulation electrode. In this test experiment a voltage was applied using a standard 9 V battery to reproduce current density modelling after tDCS.

Our results demonstrate that with our laboratory set-up the current flow measurements in the gel follow basically Ohm’s law. Fig. 1 is in line with the typical electric field heat maps where color changes indicate the typical resistance-length-dependence law which states that as the length of the material increases, resistance increases and current decreases. This is also in line with the results of modelling of the current flow through the human eye (Gall et al., 2016) and we therefore expect similar electrical field distribution when current passes through the rat’s eye.

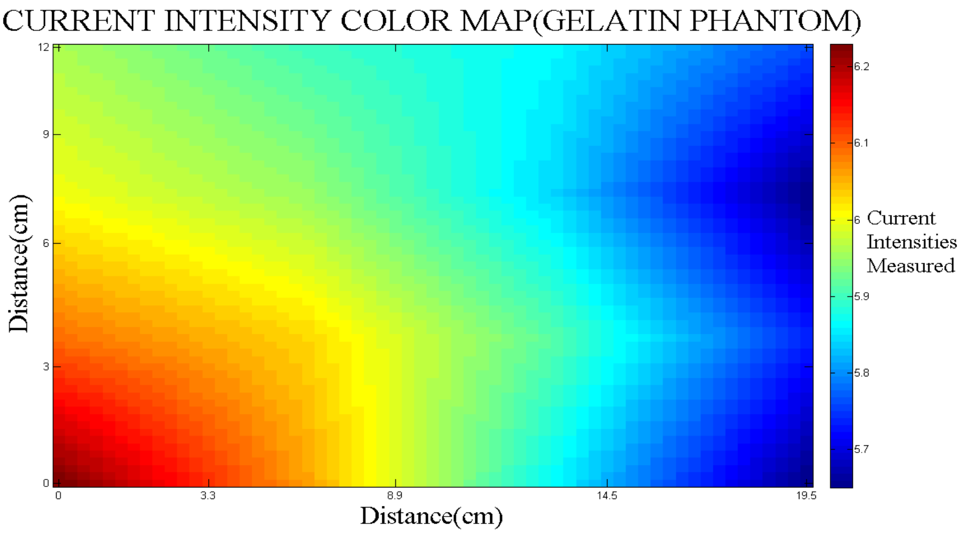

Fig. 1
This heat map from the simplified phantom model yielded similar results to what has been published from modelling approaches regarding current intensities after transcranial direct current stimulation (e.g. Jackson et al., 2016).

To address the issue of the influence of different reference electrode positions in our rat and mouse rtACS experiments, in a first approach we also used our phantom model to analyze alternating current stimulation effects. With this experimental set-up the stimulation artefacts from different electrodes placed at various distances from the stimulation electrode (which was located as described before in the left lower corner of the tray) were recorded after stimulation with 50, 100, 200 and 300 µA.

However, we did not find any difference in the shape and amplitude of the recorded signal from the different electrodes when measuring repeatedly after applying alternating currents (Fig. 2).

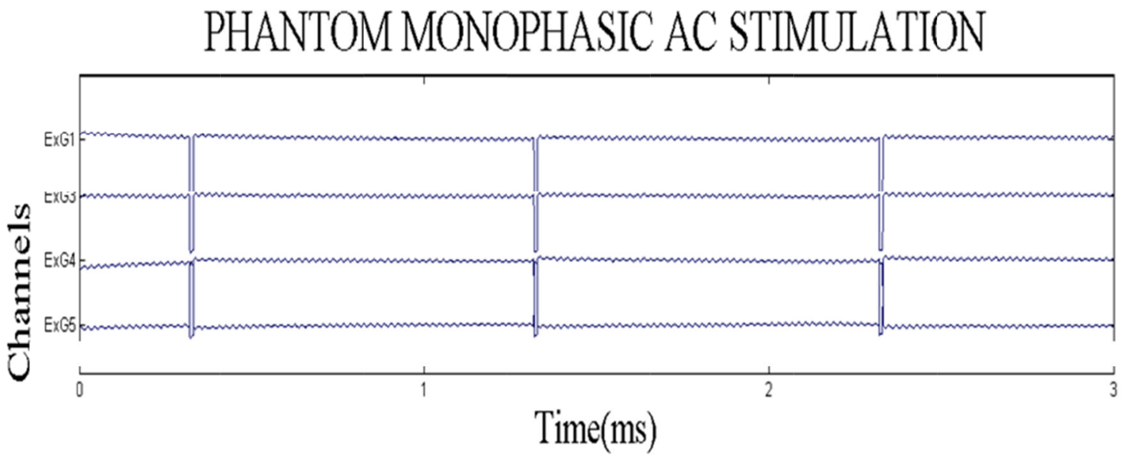

Fig. 2 shows a representative recording of the stimulation artefact traces in the gel from 3 electrodes at different positions.

However, it has to be taken into account, that the gelatin gel has a uniform resistance – and it may be therefore a good model for the rather uniform conditions of the eye, but when considering reference electrode positions in the periphery (tail, ear) the tissues are not at all uniform but have very different resistance/conductance (skin, bones, organs, fur) and different shapes (resistance also depends on the geometry). Therefore we assumed that the phantom model – although helpful for direct current density modelling in uniform tissue - may have its limitations for investigating the physics of alternating current with electrode placements in the periphery. Therefore we assumed that addressing this issue, measurements in vivo are more appropriate

Lit.:

Gall C, Schmidt S, Schittkowski MP, Antal A, Ambrus GG, Paulus W, Dannhauer M, Michalik R, Mante A, Bola M, Lux A, Kropf S, Brandt SA, Sabel BA (2016) Alternating Current Stimulation for Vision Restoration after Optic Nerve Damage: A Randomized Clinical Trial. PLoS One. 29;11(6):e0156134.

Jackson MP, Rahman A, Lafon B, Kronberg G, Ling D, Parra LC, Bikson M (2016) Animal models of transcranial direct current stimulation: Methods and mechanisms. Clinical Neurophysiology. 127: 3425–3454

Rush S, Driscoll DA (1968) Current distribution in the brain from surface electrodes. Anesth Analg. 47(6):717-23.
